# Targeted therapy of multiple liver metastases after resected solitary gastric metastasis and primary pulmonary adenocarcinoma

**DOI:** 10.18632/oncotarget.13114

**Published:** 2016-11-04

**Authors:** Ling-yu Ding, Ke-jun Liu, Zhe-long Jiang, Hai-ying Wu, Shi-xiu Wu

**Affiliations:** ^1^ Department of Medical Oncology, Hangzhou Cancer Hospital, Hangzhou, China; ^2^ Department of Medical Oncology, Sun Yat-Sen University Cancer Center, Guangzhou, China; ^3^ State Key Laboratory of Oncology in South China, Guangzhou, China; ^4^ Collaborative Innovation Center of Cancer Medicine, Guangzhou, China; ^5^ Department of Medical Oncology, Dongguan People's Hospital, Dongguan, China; ^6^ Department of Emergency, Hangzhou First People's Hospital, Nanjing Medical University, Hangzhou, China; ^7^ Department of Radiation Oncology, Hangzhou Cancer Hospital, Hangzhou, China

**Keywords:** lung cancer, gastric metastasis, surgery, liver metastases, targeted therapy

## Abstract

Gastric metastases from lung adenocarcinoma are rare and usually asymptomatic. A 61-year-old woman was referred to our department because of a right lower pulmonary mass found on a chest X-ray film in August 2012. Right lower lobectomy was performed for pulmonary adenocarcinoma. Four months later, she developed epigastric discomfort. A fluoro-deoxy-glucose positron emission tomography/computed tomography (FDG-PET/CT) scan showed a malignancy at the cardias of the stomach. A biopsy diagnosed poorly differentiated carcinoma and a gastric carcinoma was suspected. She underwent a subtotal gastrectomy and part of esophagectomy. The histologic diagnosis was metastasis from the pulmonary adenocarcinoma. She visited us again for her increasing level of carcinoembryonic antigen (CEA) after two months. FDG-PET/CT showed multiple malignant lesions in her liver, considering metastases from pulmonary origin. As she harbored activating epidermal growth factor receptor (EGFR) mutation, she received erlotinib from April, 2013. She survives 4 years after the lung resection and is still on erotinib treatment with complete response. Although gastric metastasis from lung cancer is considered a late stage of the disease, a radical resection might provide survival in solitary metastasis. Moreover, systemic therapy was emphasized after local treatment in some late stage cases.

## INTRODUCTION

The prognosis of advanced non-small-cell lung cancer (NSCLC) is poor. Metastatic sites often involved lung, liver, brain and bone, but metastases to the gastrointestinal tract are considered unusual [1–3]. Gastric involvement is even rarer and considered a late stage of the disease with a poor prognosis [2–4]. It is rarely found in clinical situations because patients are usually asymptomatic [5].

No standard treatment of gastric metastasis from the lung has yet been established. Actually only sporadic cases were reported. Most of the cases presented the significance of surgical resection either for a long-term survival or a palliative care. We report a case of isolated metachronous gastric metastasis 4 months after surgery for pulmonary adenocarcinoma, and special emphasis on follow-up treatment.

## CASE REPORT

**Figure 1:**
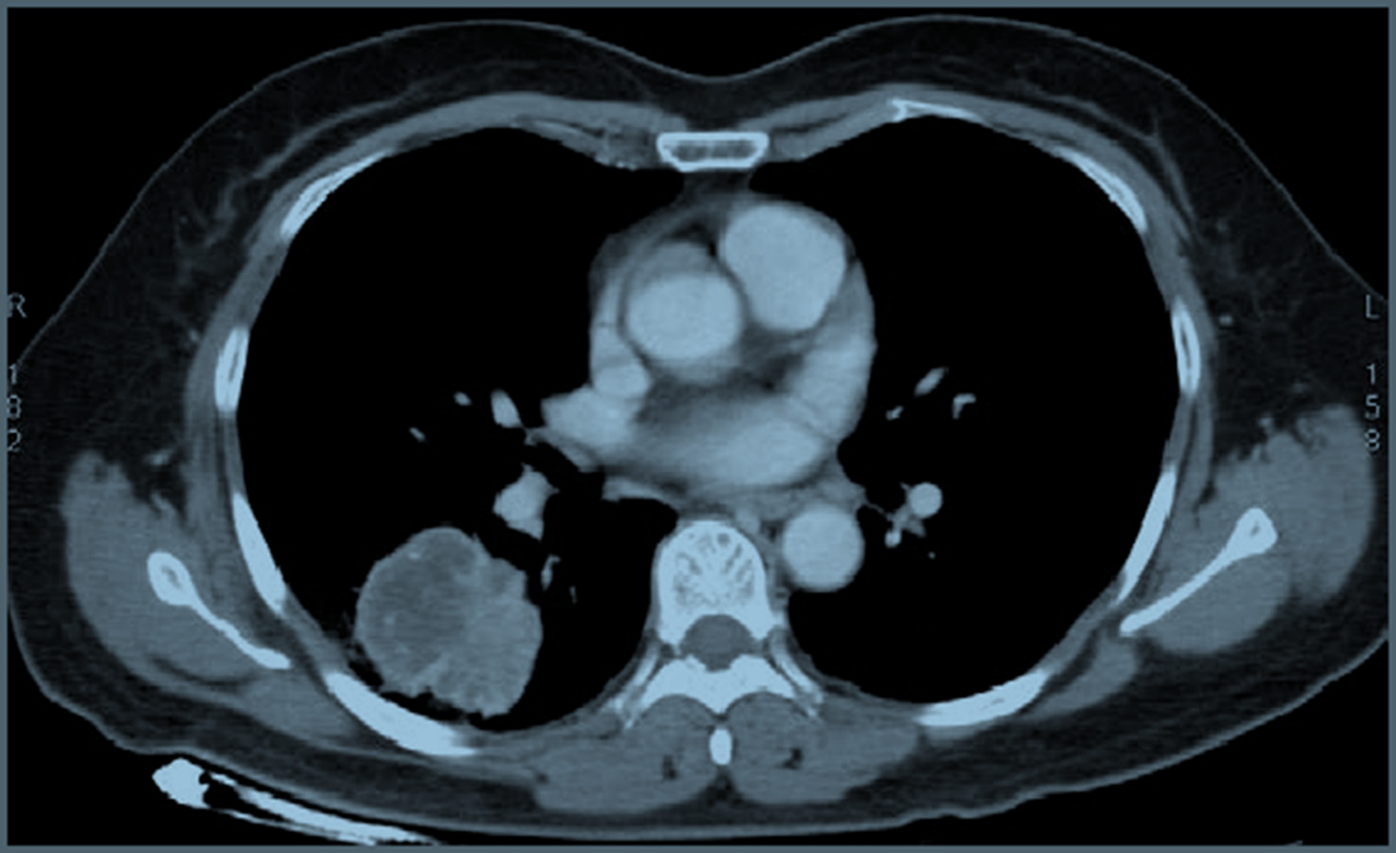
CT scan showing the mass in the right lower lung

**Figure 2:**
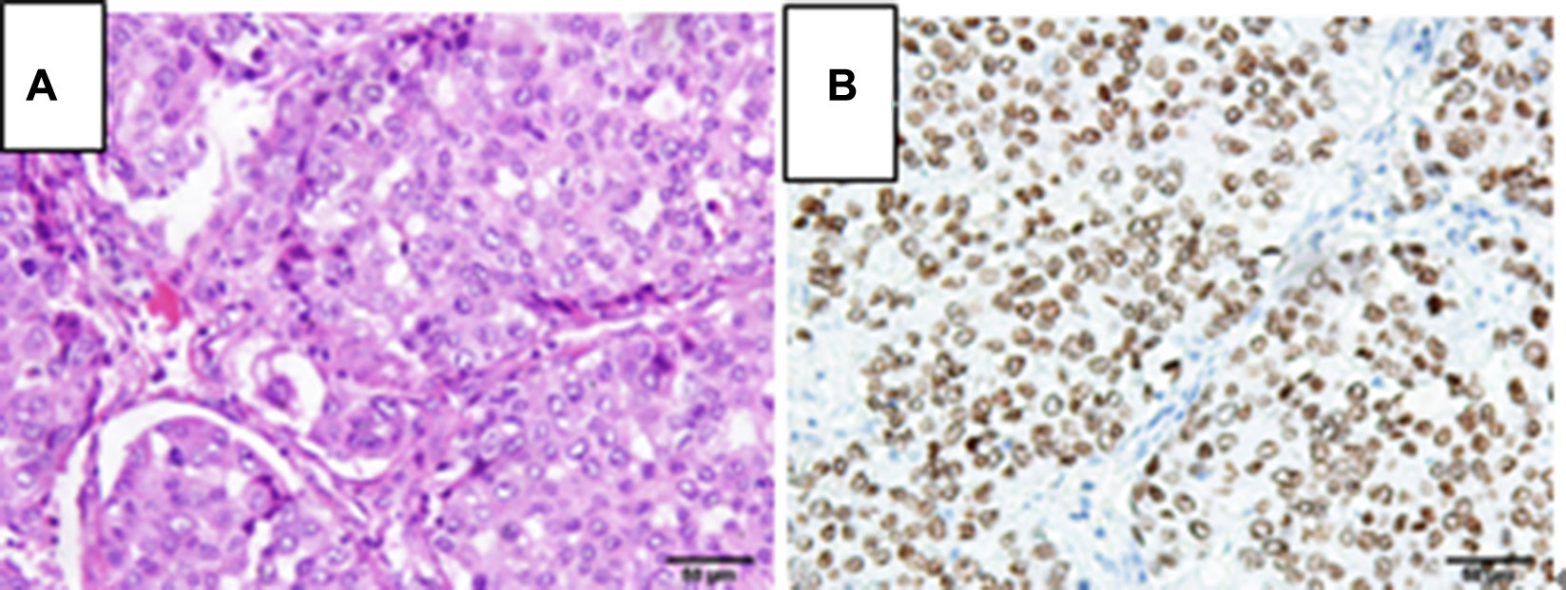
Pathological examination of the lung tumor orienting toward a poorly differentiated adenocarcinoma, (**A**) hematoxylin and eosin stain, 200×; (**B**) TTF-1 immunohistochemistry stain, 200×.

An abnormal chest x-ray shadow was detected in a 61-year-old woman in a regular check-up in August 2012. She was referred to our hospital due to a mass considered pulmonary carcinoma. The patient did not experienced any symptoms, such as cough, chest pain, fatigue, weight loss, but with carcinoembryonic antigen (CEA) 187.4 ng/ml. Bronchoscopy revealed chronic inflammation without carcinoma cell. She underwent preoperative chest and abdominal computed tomography, head magnetic resonance and bone imaging. It revealed a solitary mass measuring 4.8 cm in size without metastasis in other sites (Figure 1). In September, she underwent right lower lobectomy and mediastinal lymph node dissection. The histological diagnosis was poorly differentiated adenocarcinoma (pT2aN0M0, stage IB) (Figure 2A), with positive Thyroid Transcriptional Factor-1 (TTF-1) (Figure 2B) and Cytokeratin 7 (CK7). Mutational analysis revealed epidermal growth factor receptor (EGFR) wild type and anaplastic lymphoma kinase (ALK) negative. She was a non-smoker, but a Hepatitis B virus carrier for 21 years.

**Figure 3:**
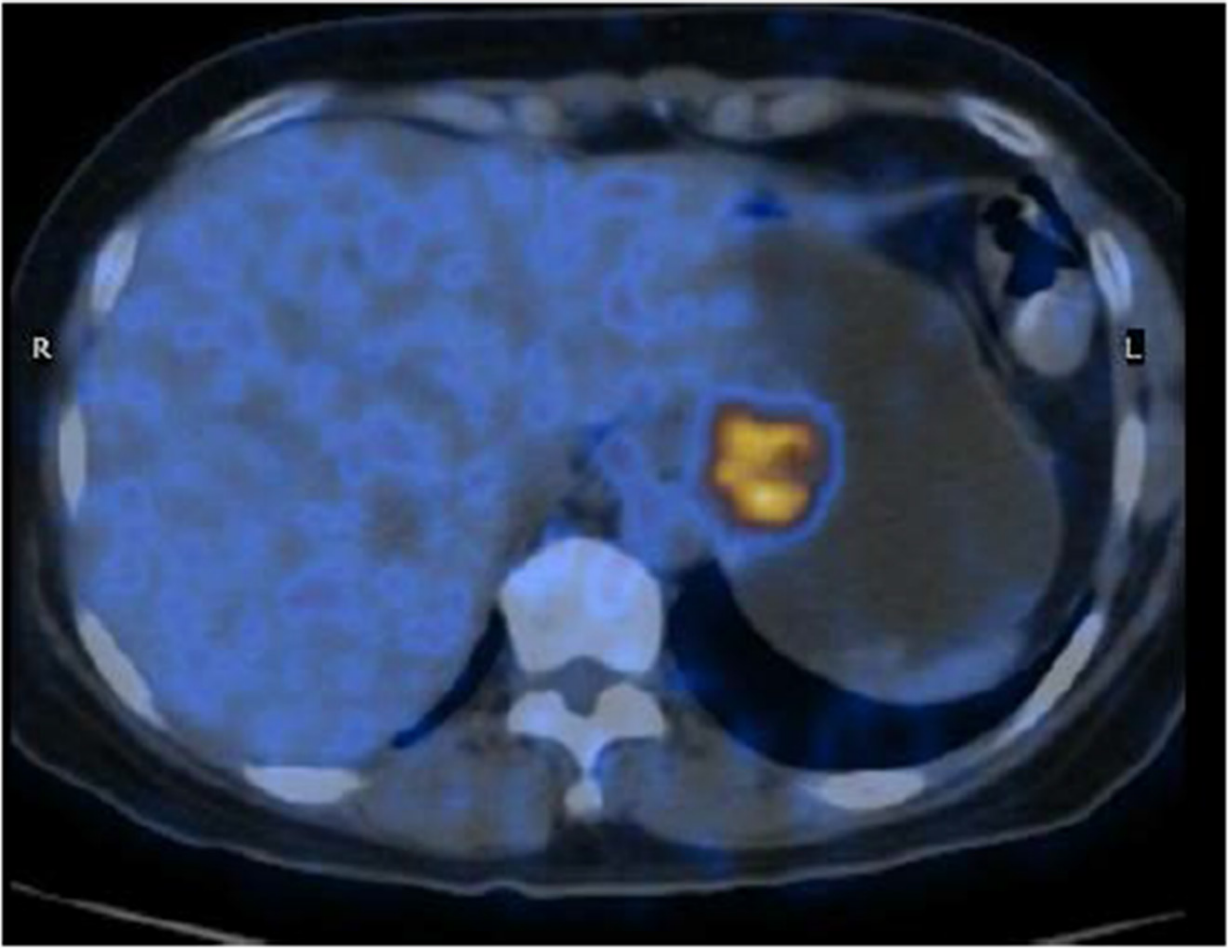
PET/CT scan showing the gastric lesion in the cardia

After the surgery, the patient did not undergo any adjuvant therapy due to her refusal. She continued the regular oncological follow-up. Four months later, she complained epigastric discomfort. CEA is still abnormal (CEA 124.2 ng/ml). Fluoro-deoxy-glucose positron emission tomography/computed tomography (FDG-PET/CT) scan confirmed a gastric malignancy (SUV19.4) at the level of the cardias with a paraesophageal lymph node metastasis (SUV11.5) (Figure 3). Subsequently, biopsy through endoscopic ultrasonography (EUS) defined the bulky lesion as a poorly differentiated carcinoma with mucosal ulceration, easy bleeding, located at the fundus, measuring 4*4 cm in diameter. The patient had no history of Helicobacter pylori infection.

**Figure 4:**
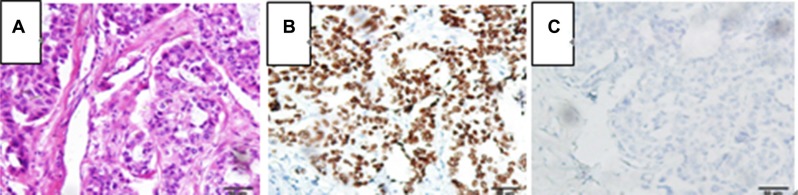
Pathological examination of the gastric tumor orienting toward a poorly differentiated adenocarcinoma metastasized from lung cancer, (**A**) hematoxylin and eosin stain, 200×; (**B**) TTF-1 immunohistochemistry stain, 200×; (**C**) CDX-2 immunohistochemistry stain, 200×.

Based on the FDG-PET/CT and EUS findings, a primary gastric carcinoma was suspected, with no evidence of advanced stage. A surgical resection was advised to her case. Finally, a subtotal gastrectomy combined with part of esophagectomy and reginal lymph node dissection was admitted. Pathology revealed poorly differentiated adenocarcinoma with serosal invasion, paracardial nodes and left gastric nodes metastasis (Figure 4A). Unexpectedly, when together with immunohistochemical (IHC) staining, it uncovered a gastric metastasis from lung cancer. IHC showed positive staining for TTF-1 (Figure 4B) and CK7 while caudal-related homeodomain transcription 2 (CDX2) negative (Figure 4C). Meanwhile, EGFR mutational analysis of gastric specimen using sequencing method was performed and revealed the presence of an exon 19 deletion in the EGFR gene. The EGFR mutational analysis of lung specimen which revealed wild type was through fluorescent quantitative polymerase chain reaction (PCR). Amplification refractory mutation system (ARMS) was used to detect EGFR mutation of lung specimen again. It turned the presence of an exon 19 deletion in the EGFR gene too. Primary lung carcinoma and metastatic gastric carcinoma both had a base-pair deletion at exon 19 (del746_A750) in the EGFR gene. Therefore, the gastric lesion was diagnosed as adenocarcinoma consistent with metastasis from primary lung carcinoma. Subsequently to the lung and gastric surgery, adjuvant chemotherapy was suggested after postoperative recovery.

**Figure 5:**
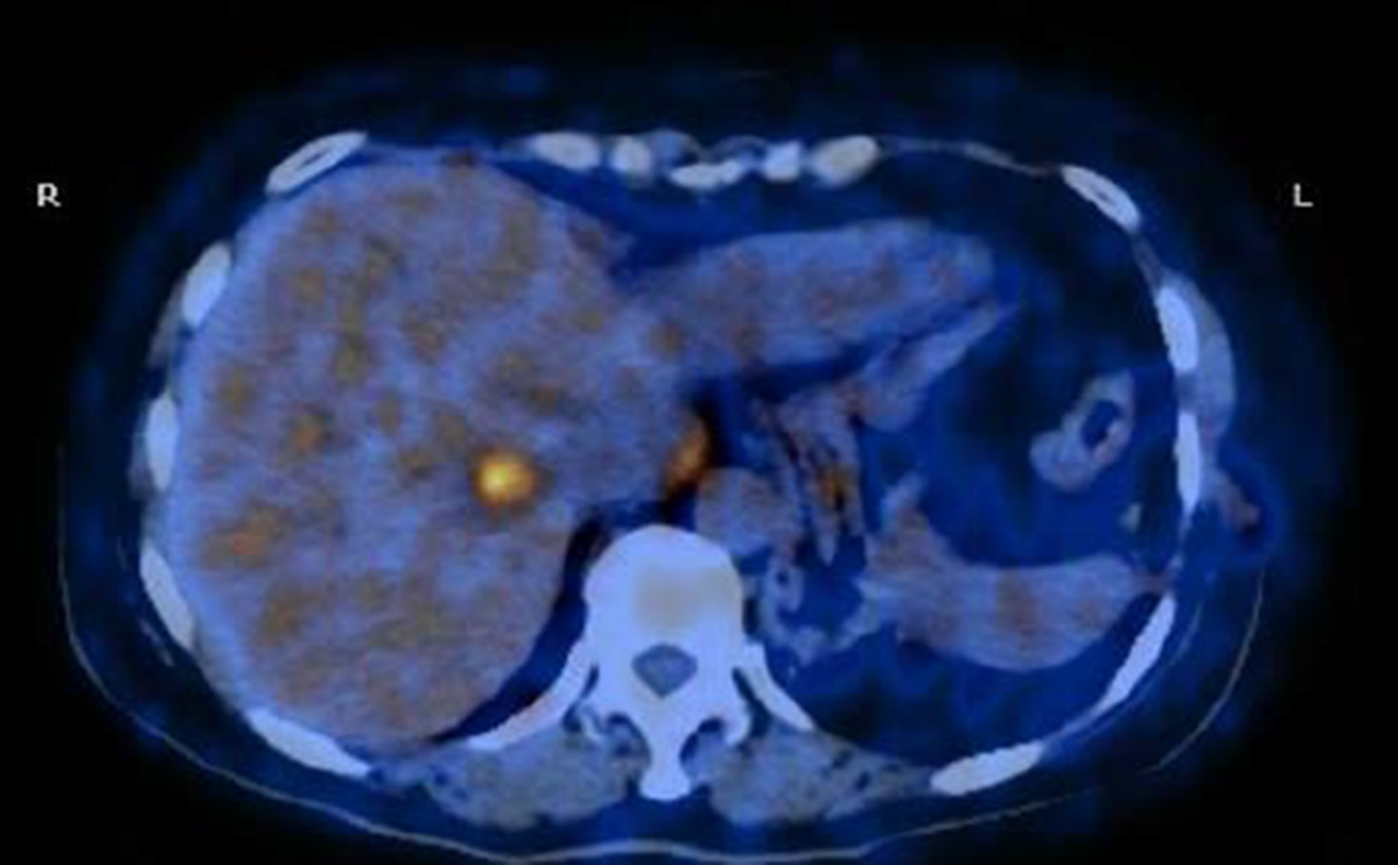
PET/CT scan showing multiple malignant lesions in the liver and suggesting liver metastases

**Figure 6:**
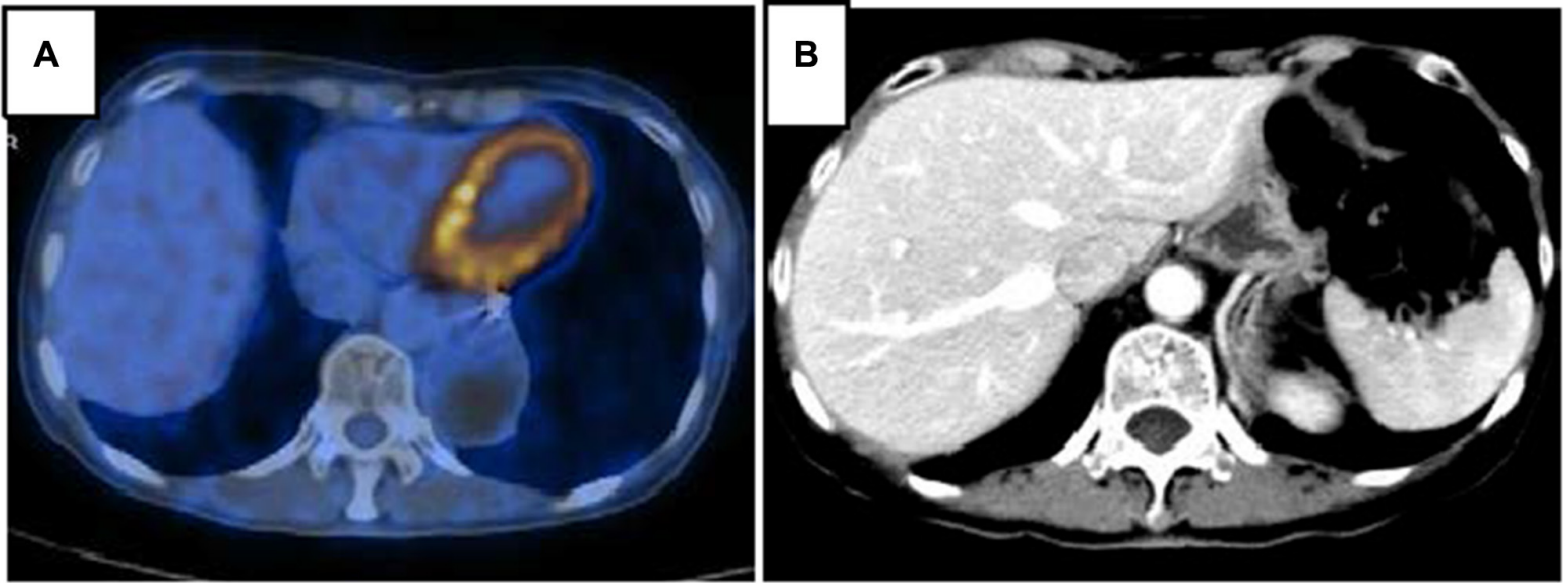
PET/CT (**A**) and CT with contrast (**B**) in follow-up care showing liver lesions disappeared after erlotinib therapy and achieved CR.

Nevertheless, two months later, the patient was referred to our medical consulting room. The level of CEA increased once again following decreasing after the gastric surgery. We performed a FDG-PET/CT due to her CEA 146 ng/ml and baseline nuclear medicine scan. The FDG-PET/CT performed on April, 2013 showed multiple malignant lesions in her liver and suggested liver metastases (Figure 5). She refused liver biopsy. Considering her medical history and imaging findings, the liver lesions were diagnosed as metastases from primary lung carcinoma. She started on oral erlotinib (150 mg, on an empty stomach) for her advanced-stage EGFR exon 19 mutation-positive lung adenocarcinoma from April, 2013. CEA in following up gradually declined to normal until September, 2013. Unexpectedly, tumour assessment by FDG-PET/ CT on 17th February, 2014 using RECIST showed a complete response (CR) (Figure 6A). During the period of erlotinib treatment, chest and abdominal CT was performed every two months and showed radiology CR (Figure 6B). Assessments of toxic effects using NCI-CTCAE showed grade 2 neutropenia and grade 2 fatigue. The patient has taken oral erlotinib for 40 months and is still going on oral erlotinib treatment. She was controlled conservatively and survived for a good quality of life.

## DISCUSSION

Lung cancer represented the leading cause of cancer death world-wide of both men and women. About 50% have distant metastasis at the time of diagnosis. The most common sites of extra-thoracic metastasis include brain, liver, adrenal glands and bone [6, 7]. Gastrointestinal tract involvement from primary lung cancer rarely occurs [2, 3, 8], especially gastric metastases [9]. Actually, gastrointestinal metastasis from lung carcinoma is expected to be higher. Possible reasons are: gastrointestinal metastases are often asymptomatic [5]; the increasing use of gastrointestinal endoscopy are performed in general hospitals; clinicians and pathologists have wealth experience in diagnose. The incidence of gastric metastasis from primary lung cancer in an autopsy series have been reported to be 0.5 to 9% [5].

In this report, the patient suffered from gastric metastasis at 4 months after lobectomy. She had a disease of stage IB lung cancer. After surgery of stage IB NSCLC, surveillance or adjuvant chemotherapy is recommended. She did not receive adjuvant chemotherapy as for her refusal. Nevertheless, she complained of epigastric discomfort and an increasing of CEA 4 months later. Then a gastric malignancy was disclosed and gastric metastasis was diagnosed postoperatively. Even though most patients with gastric metastasis are asymptomatic, they will suffered from symptoms of abdominal pain, chronic bleeding, nausea and vomiting [8]. Hence, complaint and laboratory tests cannot be ignored in clinical practice. Adjuvant chemotherapy is not recommended for routine use for patients with completely resected stage IB NSCLC [10], but significantly improves survival for patients who had tumors ≥ 4.0 cm even from exploratory analysis [11]. Maybe, this patient could benefit from adjuvant chemotherapy.

Most extrathoracic recurrences after complete NCSLC resection are multiple and disseminated, and destined to a poor prognosis [12]. Patients of disseminated recurrences usually need to be treated with systemic chemotherapy for a palliative purpose, while operative treatments are not indicated. However, some investigators reported long-term survival after surgical resection in selected patients [9, 13]. The characteristic of gastric metastasis is exactly alike. Metastases to the stomach become symptomatic after a considerable growth. Surgical resection may be indicated when complications such as bleeding, perforation and obstruction occur, even if the disease is disseminated. This case was a solitary metachronous gastric metastasis from pulmonary adenocarcinoma. A radical gastrectomy was advised to her. In terms of FDG-PET/CT and pathologic finding, it's usually difficult to diagnose a metastasis from a primary lung cancer to the stomach, especially when the histology is adenocarcinoma. But immunohistochemical staining can be used to differentiate primary pulmonary adenocarcinoma from gastrointestinal adenocarcinoma. While positive staining for CK7 is consistent with either agastrointestinal or pulmonary origin of the tumor, TTF-1 positivity is limited to tumors of the thyroid and lung. Given that no tumor was found in the patient’s thyroid gland, the patient’s gastric lesion was metastasized from the lung. Moreover, negative staining for CDX2 supported the gastric lesion was not primary gastrointestinal tumor. Sometimes, gene analysis, such as next generation sequencing (NGS) analysis, may help distinguish origin from metastasis. In this case, the gastrectomy specimen expressed CK7, TTF-1 and harbored EGFR-activating mutation, in accordance with the lobectomy specimen.

In the regular surveillance after lobectomy, for a solitary metastatic lesion in the brain or adrenal gland, local treatment is advised. Nevertheless, literature data on surgical treatment of single gastric metastasis is scant, not to mention the subsequent treatment. In this case, the patient underwent gastrectomy for the solitary gastric metastasis. So the question is: what's the next step for her? The small number of case reports had no systematic treatments after surgical intervention of both primary and metastatic tumors [2, 4, 14]. In view of the short interval between the diagnosis of lung cancer and the diagnosis of gastric metastasis, adjuvant chemotherapy with cisplatin and pemetrexed was suggested to her. Nevertheless, the patient thought that she was too asthenic to receive adjuvant chemotherapy. Therefore, she refused chemotherapy and went home to have a postoperative recovery.

Unfortunately, she relapsed again with multiple liver lesions soon! Patients with advanced NSCLC known to carry an activating mutation in the EGFR gene should be treated with an EGFR-tyrosine kinase inhibitor (EGFR-TKI). Different randomized phase III trials described consistent data [15–17]. The patient was started on first-line therapy of oral erlotinib for her EGFR sensitive mutation. Trials of erlotinib as first-line treatment of advanced NSCLC showed a median progression-free survival (PFS) of 10–13 months [15, 16]. In the case, the patient experienced a PFS of 40 months with a CR. She has survived more than 4 years without any further signs of recurrence and may result in a cure.

We retrospectly reviewed this case and proposed that solitary gastric metastasectomy following complete pulmonary carcinoma resection may be indicated. In addition, systemic treatment may be indicated after surgical resection of a solitary NSCLC recurrence. Particularly when patients harbor activating mutations in the EGFR gene or chromosomal rearrangements involving the ALK gene, molecular targeted TKIs perhaps provide clinical benefit with low toxicity. More cases are necessary to evaluate the effectiveness of resection of gastric metastasis and adjuvant therapies after resection of a solitary NSCLC recurrence.
